# The radical SAM enzyme NirJ cleaves off two propionate side chains with the release of acrylate during heme *d*_1_ biosynthesis

**DOI:** 10.1111/febs.70105

**Published:** 2025-05-02

**Authors:** Heike Meyer, Maren H. Hoock, Kai Zwara, Sören Jahn, Volker Schünemann, Gunhild Layer

**Affiliations:** ^1^ Pharmazeutische Biologie, Institut für Pharmazeutische Wissenschaften Albert‐Ludwigs‐Universität Freiburg Germany; ^2^ Fachbereich Physik RPTU Kaiserslautern‐Landau Germany; ^3^ Pharmazeutische und Medizinische Chemie, Institut für Pharmazeutische Wissenschaften Albert‐Ludwigs‐Universität Freiburg Germany

**Keywords:** heme *d*_1_ biosynthesis, iron–sulfur cluster, Mössbauer spectroscopy, radical SAM, tetrapyrroles

## Abstract

Heme *d*_1_ is an iron‐containing, modified tetrapyrrole that serves as an essential prosthetic group in cytochrome *cd*_1_ nitrite reductases. The biosynthesis of heme *d*_1_ from the precursor siroheme requires three or four enzymatic steps, including the removal of two propionate side chains, the latter being catalyzed by the radical SAM enzyme NirJ. Although the removal of the propionate side chains by NirJ has been shown previously, several aspects of NirJ catalysis remained elusive, including the type of its auxiliary iron–sulfur cluster as well as the identity of the cleavage byproduct and the actual product of the NirJ reaction. Here, we demonstrate by Mössbauer spectroscopy that NirJ contains a [4Fe‐4S] cluster ligated by cysteine residues as its auxiliary cluster. We show that acrylate is released during the NirJ reaction as the cleavage byproduct, as observed by HPLC‐UV and HPLC–MS analysis of enzyme activity assay mixtures after derivatization. Finally, we provide strong evidence from HPLC‐UV/Vis and HPLC‐MS analysis that the NirJ reaction product contains methylene groups at positions C3 and C8 of the tetrapyrrole macrocycle. Based on these results, we propose a revised version of the NirJ reaction mechanism, including a potential role of the auxiliary iron–sulfur cluster as an electron donor for radical quenching.

## Graphical abstract

The radical SAM enzyme NirJ is responsible for the removal of two propionate side chains from its substrate didecarboxy‐siroheme during heme *d*_1_ biosynthesis. The cleavage products of the NirJ reaction were not known so far. In this study, it is shown that acrylate is formed as the reaction byproduct and that the resulting tetrapyrrole product carries methylene groups after the removal of the propionate groups.

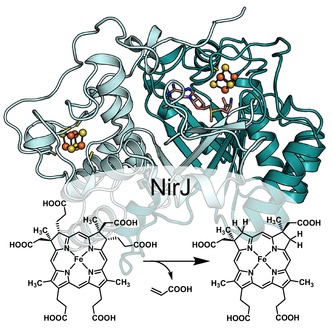

**Abbreviations**
5′‐dA: 5′‐deoxyadenosine
5′‐dA˙: 5′‐deoxyadenosyl radical
aux: auxiliary
CV: column volume
DDSH: 12,18‐didecarboxy‐siroheme
DT: dithionite
ESI: electrospray ionization
HPLC: high‐performance liquid chromatography
IMAC: immobilized metal ion affinity chromatography
LC: liquid chromatography
MS: mass spectrometry
MS/MS: tandem mass spectrometry
NirJ‐1CM: NirJ variant lacking the RS‐cluster
RS: radical SAM
SAM: *S*‐adenosyl‐l‐methionine
UV/Vis: ultraviolet/visible
XIC: extracted ion chromatogram

## Introduction

**Fig. 1:**
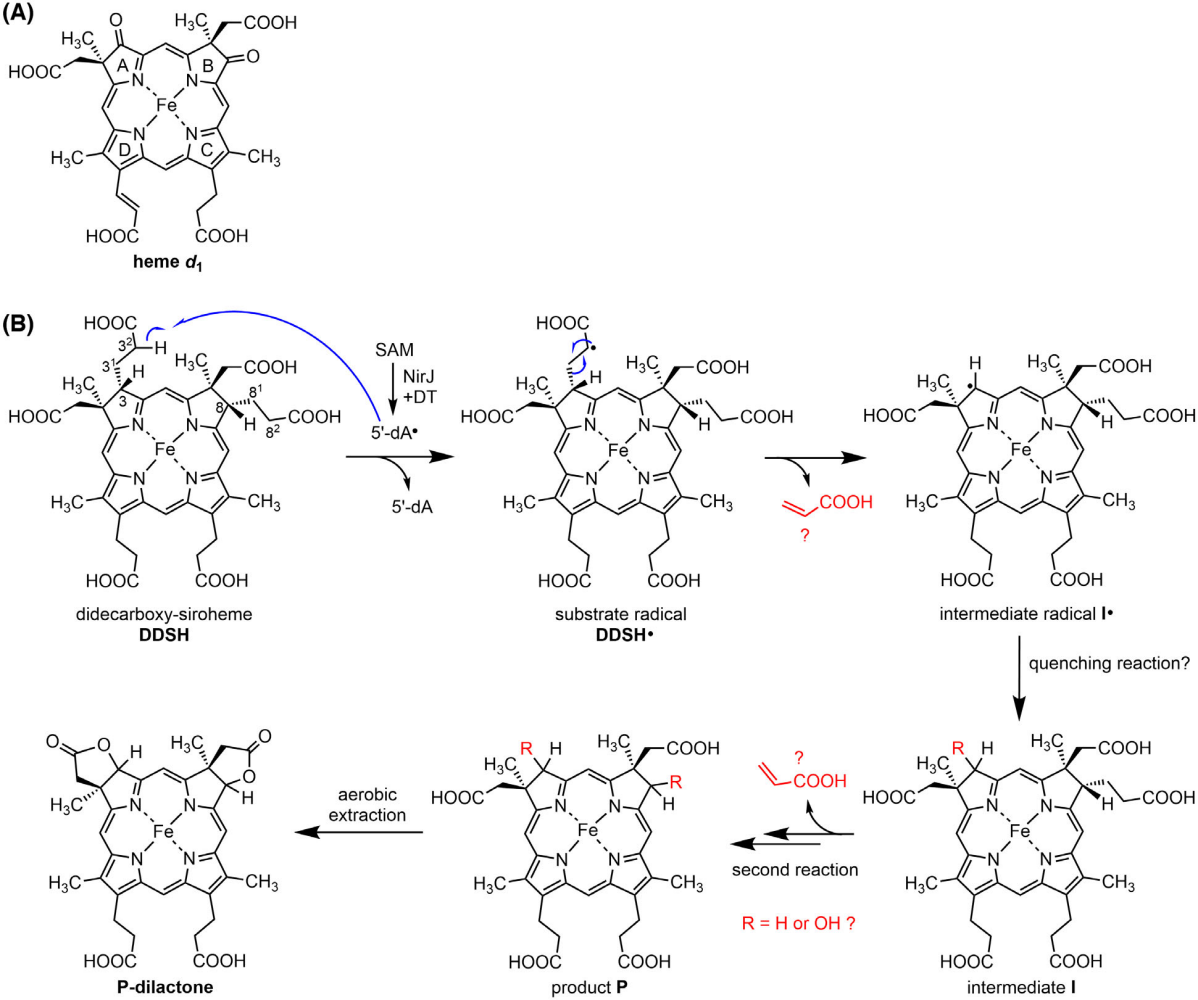
Structure of heme *d*_1_ and proposed reaction mechanism for NirJ. (A) Chemical structure of heme *d*_1_ with the pyrrole rings denoted A–D. (B) Potential reaction mechanism for NirJ as proposed by Boss *et al*. [6]. Numbering of the propionate side chains of pyrrole rings A and B is shown. Parts of the mechanism that lack experimental evidence so far are shown in red. The figure was generated using chemdraw (Revvity Signals Software Inc., Waltham, MA, USA). 5′‐dA, 5′‐deoxyadenosine; 5′‐dA˙, 5′‐deoxyadenosyl radical; DDSH, didecarboxy‐siroheme; DT, dithionite; I, intermediate; P, product; SAM, *S*‐adenosyl‐l‐methionine.

Denitrifying bacteria play an important role in the global biogeochemical nitrogen cycle by converting nitrate into molecular nitrogen in a four‐step enzymatic process [1]. For the second step of denitrification, the reduction of nitrite to nitrogen monoxide, many of these bacteria use the cytochrome *cd*_1_ nitrite reductase NirS [2], which contains heme *d*_1_ as the catalytically active cofactor [3]. Heme *d*_1_ is a modified, iron‐containing tetrapyrrole (Fig. 1A) that is derived from the biosynthetic precursor siroheme [4]. In the course of heme *d*_1_ biosynthesis, the siroheme decarboxylase NirDLGH converts siroheme into 12,18‐didecarboxy‐siroheme (DDSH) [4, 5]. In the next step, the two propionate side chains at pyrrole rings A and B of DDSH are removed, which is accomplished by the enzyme NirJ [6]. In the following, the two keto functions at pyrrole rings A and B must be introduced via an as yet unknown reaction. Also, the enzyme responsible for this step is not known yet. In the final step of heme *d*_1_ biosynthesis, NirN catalyzes the dehydrogenation of the propionate side chain at pyrrole ring D to an acrylate side chain [7].

The heme *d*_1_ biosynthesis enzyme NirJ belongs to the family of radical *S*‐adenosyl‐l‐methionine (SAM) enzymes, all of which contain a [4Fe‐4S] cluster ligated by three cysteine residues [8]. Coordination of the fourth, non‐cysteine ligated iron ion of this radical SAM (RS) cluster is provided by SAM through the amino and carboxylate groups of its methionine moiety [9, 10]. The initial reaction steps of all radical SAM enzymes consist of (a) the reduction of the RS‐cluster to the 1+ state, (b) electron transfer to the cluster‐bound SAM, (c) homolytic cleavage of the S‐C5′ bond of SAM yielding methionine and an organometallic intermediate Ω [11, 12], (d) liberation of the reactive 5′‐deoxyadenosyl radical (5′‐dA˙) and (e) hydrogen atom abstraction by the 5′‐dA˙ from the substrate resulting in the formation of a substrate radical and 5′‐deoxyadenosine (5′‐dA) [13]. The following reaction steps converting the substrate radical into the reaction product are different for each individual radical SAM enzyme. Within the radical SAM enzyme family, several subfamilies were defined, to which the members can be allocated either by the type of catalyzed reaction or by the presence of characteristic domains and additional cofactors [14]. One of these subfamilies is the so‐called SPASM/twitch‐domain family, the members of which carry either one or two additional (auxiliary) iron–sulfur clusters besides the RS‐cluster [15].

In previous studies with NirJ, it was shown that the enzyme contains the characteristic RS‐cluster, to which SAM binds as the prerequisite for the initiation of radical catalysis [16]. Furthermore, it was shown that NirJ contains one auxiliary (aux) iron–sulfur cluster, most likely coordinated by cysteine residues of the conserved sequence motif CX_2_CX_5_CX_20–21_C present in the C‐terminal domain of the protein [6]. Indeed, the C‐terminal end of NirJ is predicted to adopt a SPASM/twitch‐domain fold, placing the enzyme into this radical SAM subfamily (https://www.ebi.ac.uk/interpro/protein/UniProt/A8LLZ7/). Although the presence of one aux‐cluster in NirJ was shown, the exact cluster type ([4Fe‐4S] or [2Fe‐2S]) as well as the cluster ligation (three or four cysteine ligands) of the aux‐cluster remained elusive.

As described above and shown previously, NirJ catalyzes the removal of two propionate side chains of DDSH with concomitant formation of 5′‐dA as a co‐product. A potential mechanism was proposed for this radical‐based reaction (Fig. 1B) [6]. In order to initiate side chain removal, the 5′‐dA˙ is proposed to abstract a hydrogen atom from either C3^2^ or C8^2^ depending on which of the propionate side chains is removed first. The proposed fragmentation reaction of the substrate radical might result in the release of acrylate as the leaving group and the formation of an intermediate radical. Then, a final radical quenching step yields the product of the first side chain removal reaction. The removal of the second propionate side chain most likely proceeds in a similar fashion. At present, several aspects of this mechanism are purely hypothetical and require experimental testing. For example, the nature of the leaving group as well as the actual reaction product are not known (Fig. 1B). The identity of the reaction product remained elusive due to the proposed lactone formation during the aerobic product extraction procedure [6].

In this study, we aimed to clarify the cofactor composition of NirJ and to determine the nature of the leaving group and the product of the NirJ reaction. Using Mössbauer spectroscopy, we show that the aux‐cluster of NirJ is a [4Fe‐4S] cluster coordinated by four cysteine residues. The leaving group of the propionate side chain cleavage reaction is identified as acrylate, as shown by LC–MS analysis after chemical derivatization. Finally, we present strong evidence for the formation of a reaction product carrying methylene groups at positions C3 and C8 as a result of propionate side chain removal catalyzed by NirJ.

## Results

### NirJ contains an auxiliary [4Fe‐4S] cluster

**Fig. 2:**
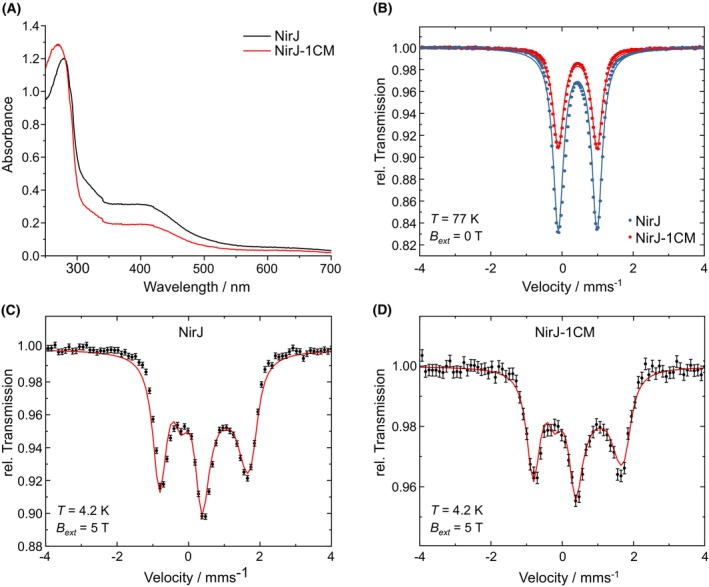
Characterization of the auxiliary iron–sulfur cluster of NirJ. (A) UV/Vis absorption spectra of wild‐type NirJ and the variant NirJ‐1CM lacking the RS‐cluster. (B) Mössbauer spectra of wild‐type NirJ and the variant NirJ‐1CM measured at 77 K without external magnetic field. (C, D) Mössbauer spectra of wild‐type NirJ (C) and the variant NirJ‐1CM (D) measured at 4.2 K and *B*_ext_ = 5 T. Dots represent the experimental data, solid lines represent the simulated spectra with the parameters given in the main text. The graphs were generated with originpro (Version 2024; OriginLab Corporation, Northampton, MA, USA) and the figure was assembled using inkscape (https://inkscape.org). NirJ‐1CM, NirJ variant lacking the RS‐cluster.

In order to clarify the nature and coordination of the aux‐cluster of NirJ, Mössbauer spectroscopy was employed. For this purpose, recombinant wild‐type NirJ and a NirJ variant lacking the RS‐cluster (NirJ‐1CM) [6] were produced in minimal medium containing ^57^FeCl_3_ for the *in vivo* incorporation of the ^57^Fe isotope into the iron–sulfur clusters of the enzymes. For both enzymes, wild type and variant, the production was done without the coproduction of DDSH, in order to obtain preparations without an iron‐containing tetrapyrrole. After protein purification under anaerobic conditions, UV/Vis absorption spectra were recorded for the ^57^Fe‐containing proteins (Fig. 2A). Mössbauer spectra measured at 77 K and at 4.2 K in a high external magnetic field are shown in Fig. 2B–D. The UV/Vis absorption spectra show a broad absorption band around 400 nm indicating the presence of [4Fe‐4S] clusters in both proteins. However, the absorption intensity of this band for the NirJ‐1CM variant is only about half of that for wild‐type NirJ. The determination of the iron content of the purified proteins revealed 3.1 mol iron per mol NirJ wild type and 1.5 mol iron per mol NirJ‐1CM indicating incomplete cluster incorporation during recombinant protein production in *Escherichia coli* as reported previously [6]. Although the iron–sulfur cluster content of the NirJ proteins could be improved by chemical reconstitution [6], we chose intentionally to use the as‐purified proteins for Mössbauer spectroscopy in order to avoid any reconstitution artifacts, especially regarding the type of the auxiliary cluster. The Mössbauer spectra measured at 77 K exhibit symmetric doublets for both proteins with identical isomer shift values of δ = 0.44 mm·s^−1^ and identical quadrupole splittings of Δ*E*_Q_ = 1.09 mm·s^−1^. These values suggest the presence of [4Fe‐4S]^2+^ clusters in both proteins. The experimental area obtained by integration of the Mössbauer spectrum (0.09) of NirJ‐1CM is half of that of wild‐type NirJ (0.18) in agreement with the UV/Vis absorption spectra and the iron content of the proteins. Importantly, the presence of diamagnetic [4Fe‐4S]^2+^ clusters was confirmed by the Mössbauer spectra measured at 4.2 K and an external magnetic field of *B*_ext_ = 5.0 T. For both proteins, the respective spectrum could be simulated with a diamagnetic component with identical parameters (δ = 0.44 mm·s^−1^, Δ*E*_Q_ = 1.20 mm·s^−1^, line width at half maximum Γ = 0.43 mm·s^−1^, asymmetry parameter η = 0.0). Altogether, these experiments show that wild‐type NirJ contains two [4Fe‐4S] clusters. The NirJ‐1CM variant still carries the auxiliary [4Fe‐4S] cluster, which is coordinated by four cysteine residues based on the fact that the Mössbauer spectra could be simulated using a single component with parameters that are characteristic for cysteine ligation [17, 18].

### Acrylate is the byproduct of the NirJ reaction

**Fig. 3:**
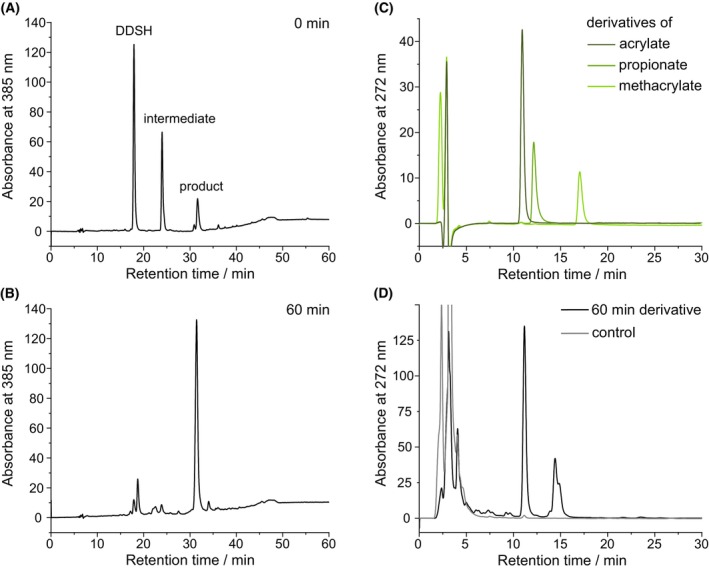
HPLC‐UV/Vis analysis of NirJ activity assays. (A, B) HPLC‐UV/Vis analysis of tetrapyrroles present in NirJ reaction mixtures (200 μm NirJ‐DDSH complex, 2 mm SAM, 2 mm DT, 1 mm NaNO_2_) after 0 min (A) and 60 min (B) of incubation. Didecarboxy‐siroheme (DDSH) elutes at 17.9 min, the reaction intermediate at 23.9 min and the reaction product at 31.4 min as indicated. (C) HPLC‐UV analysis of the short‐chain carboxylic acid standards acrylate, propionate, and methacrylate after derivatization to the corresponding *N*‐methylbenzylamine derivatives. (D) HPLC‐UV analysis of the NirJ activity assay mixture (60 min, same mixture as in A and B) for the presence of a short‐chain carboxylic acid byproduct after derivatization. A derivatized control reaction lacking NirJ is shown in gray. The substance eluting at about 14.4 min was not further investigated. The graphs were generated with originpro (Version 2024; OriginLab Corporation) and the figure was assembled using inkscape. DDSH, didecarboxy‐siroheme.

**Fig. 4:**
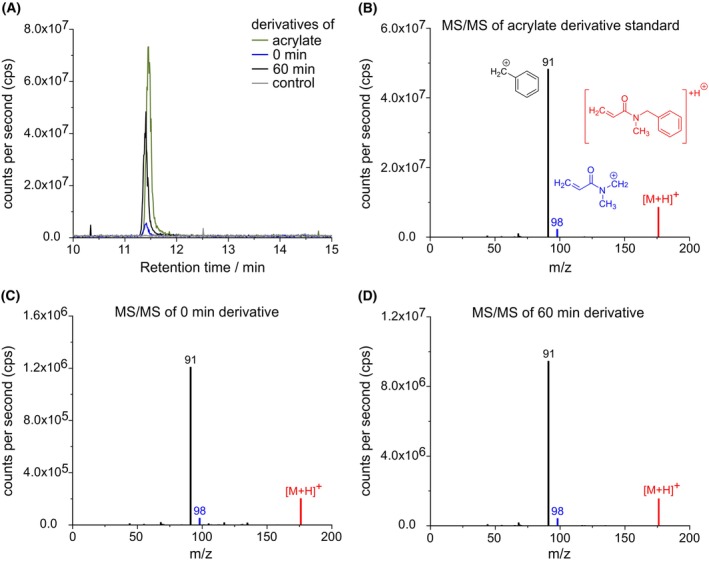
Identification of the NirJ cleavage byproduct as acrylate. (A) Extracted ion chromatograms at *m/z* of 176 of samples containing the *N*‐methylbenzylamine derivatives of the acrylate standard, the cleavage byproduct in NirJ activity assays (380 μm NirJ‐DDSH complex, 3.8 mm SAM, 3.8 mm DT, 1 mm NaNO_2_) after 0 and 60 min incubation and of a control reaction lacking NirJ. (B–D) MS/MS spectra of the samples containing the *N*‐methylbenzylamine derivatives of the acrylate standard (B) and the cleavage product in NirJ activity assays after 0 (C) and 60 min (D) incubation. The [M + H]^+^ precursor ion is highlighted in red. Chemical structures of the precursor ion with *m/z* 176 as well as the product ions with *m/z* 98 (blue) and *m/z* 91 (black) are shown in (B). The graphs were generated with originpro (Version 2024; OriginLab Corporation) and the figure was assembled using inkscape. MS/MS, tandem mass spectrometry.

An important aim of this study was to identify the cleavage byproduct that is released during the NirJ reaction (Fig. 1B). The most likely byproducts were either propionate or acrylate. In order to identify the actual NirJ byproduct, enzyme activity assays were performed. For NirJ activity assays, the enzyme was purified in complex with its substrate DDSH, and the NirJ‐DDSH complex was used without further addition of substrate. Therefore, the assays represent single turnover reactions. After incubation of the reaction mixtures, they were analyzed by HPLC‐UV/Vis for the formation of the tetrapyrrolic product as well as the cleavage byproduct. For easy separation and detection of the cleavage byproduct, a derivatization reaction was performed prior to product extraction and HPLC analysis. Therefore, the cleavage byproduct was detected as the *N*‐methylbenzylamine derivative. For the enzyme activity assay, the purified NirJ‐DDSH complex was incubated with SAM and sodium dithionite (DT) and the reaction was stopped immediately after mixing all components (0 min) and after 60 min of incubation. The HPLC‐UV/Vis analysis of the extracted tetrapyrroles is shown in Fig. 3A,B. At the start of the reaction, the substrate DDSH, intermediate I, and product P, all in their dilactone form, are observed at retention times of 17.9, 23.9, and 31.4 min, respectively. The intermediate and product detected at the start of the reaction were not formed *in vitro* within the time of mixing, but were rather produced *in vivo* during NirJ production in *E. coli* and were copurified bound to the enzyme, as previously reported [6] After 60 min of incubation, the substrate DDSH and the intermediate I were almost completely consumed, and the major peak in the HPLC chromatogram corresponds to the reaction product of the NirJ reaction, as also previously described by Boss *et al*. [6]. In Fig. 3D, the HPLC‐UV analysis of the derivatized cleavage byproduct of the same activity assay mixture after 60‐min incubation is shown. For comparison, the analysis of the derivatized standards acrylate, propionate, and methacrylate are depicted in Fig. 3C. The HPLC chromatogram of the derivatized assay mixture shows two peaks. The retention time of the peak at 11.1 min corresponds to the retention time of the derivatized acrylate standard, suggesting the formation of acrylate as the cleavage byproduct. The retention time of the second peak at 14.4 min does not correspond to any of the standards. Since acrylate is prone to polymerization, this peak might represent a derivative of dimerized acrylate; however, this was not further investigated. As a control, an assay mixture lacking the NirJ‐DDSH complex was incubated with SAM and DT for 60 min and subsequently subjected to derivatization and extraction. For this sample, the HPLC chromatogram does not exhibit any peaks at retention times corresponding to the cleavage byproduct, showing that the formation of the potential acrylate (derivative) depends on the presence of NirJ. Finally, in order to unambiguously identify the observed cleavage byproduct as acrylate, NirJ assay mixtures (0 and 60 min) were analyzed by HPLC‐MS after derivatization. The control reaction lacking NirJ‐DDSH as well as derivatized acrylate as a standard were similarly analyzed (Fig. 4). The extracted ion chromatogram (*m/z* 176, positive ion mode) of the sample containing the acrylate derivative standard (calculated mass 175.1 Da) exhibits a single peak at a retention time of 11.4 min (Fig. 4A). The corresponding MS/MS spectrum of the 176 Da precursor ion ([M + H]^+^) shows a characteristic fragmentation pattern with fragments of *m/z* 98 and *m/z* 91 (Fig. 4B). The extracted ion chromatograms of the derivatized activity assay samples (0 and 60 min) each exhibit a peak at the same retention time as that of the standard (Fig. 4A). Moreover, the corresponding MS/MS spectra are identical to that of the acrylate derivative standard (Fig. 4C,D). In contrast, no peak is observed in the extracted ion chromatogram of the derivatized assay mixture lacking NirJ‐DDSH. Based on these results, we conclude that acrylate is cleaved off as the byproduct during the NirJ reaction.

### The propionate side chains are replaced by hydrogen during the NirJ reaction

**Fig. 5:**
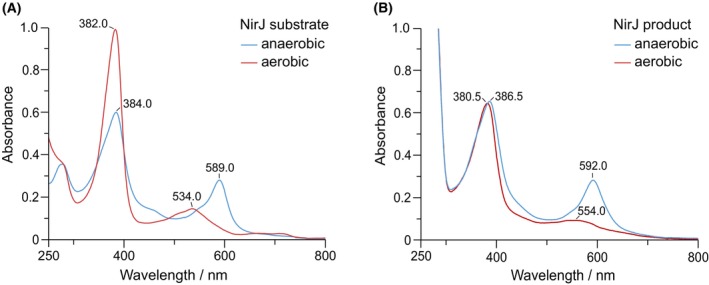
UV/Vis absorption spectra of NirJ substrate and product. (A) UV/Vis spectra of the NirJ substrate isolated by heat denaturation of the protein under anaerobic conditions (blue) and after exposure to air (red). (B) UV/Vis spectra of the NirJ reaction product isolated by heat denaturation of the protein under anaerobic conditions (blue) and after exposure to air (red). Absorption maxima are labeled, respectively. The graphs were generated with originpro (Version 2024; OriginLab Corporation) and the figure was assembled using inkscape.

So far, the actual NirJ reaction product that is formed after propionate side chain removal by quenching of the intermediate radical is not known. In previous studies, the identification of the reaction product was hampered by the fact that the acetate side chains on pyrrole rings A and B form lactones under aerobic conditions, as shown by mass spectrometry [6]. Lactone formation was also indicated by a color change of the tetrapyrroles. The substrate DDSH and the NirJ reaction product both exhibited a bluish color after anaerobic extraction from NirJ by heat denaturation of the protein. The corresponding UV/Vis spectra (Fig. 5) display absorption maxima at 384 and 589 nm for DDSH and at 386.5 and 592 nm for the NirJ reaction product. Upon exposure to air, the tetrapyrrole solutions turned red and the UV/Vis spectra changed. For DDSH, the absorption maxima were now at 382 and 534 nm and for the NirJ product at 380.5 and around 554 nm (Fig. 5).

**Fig. 6:**
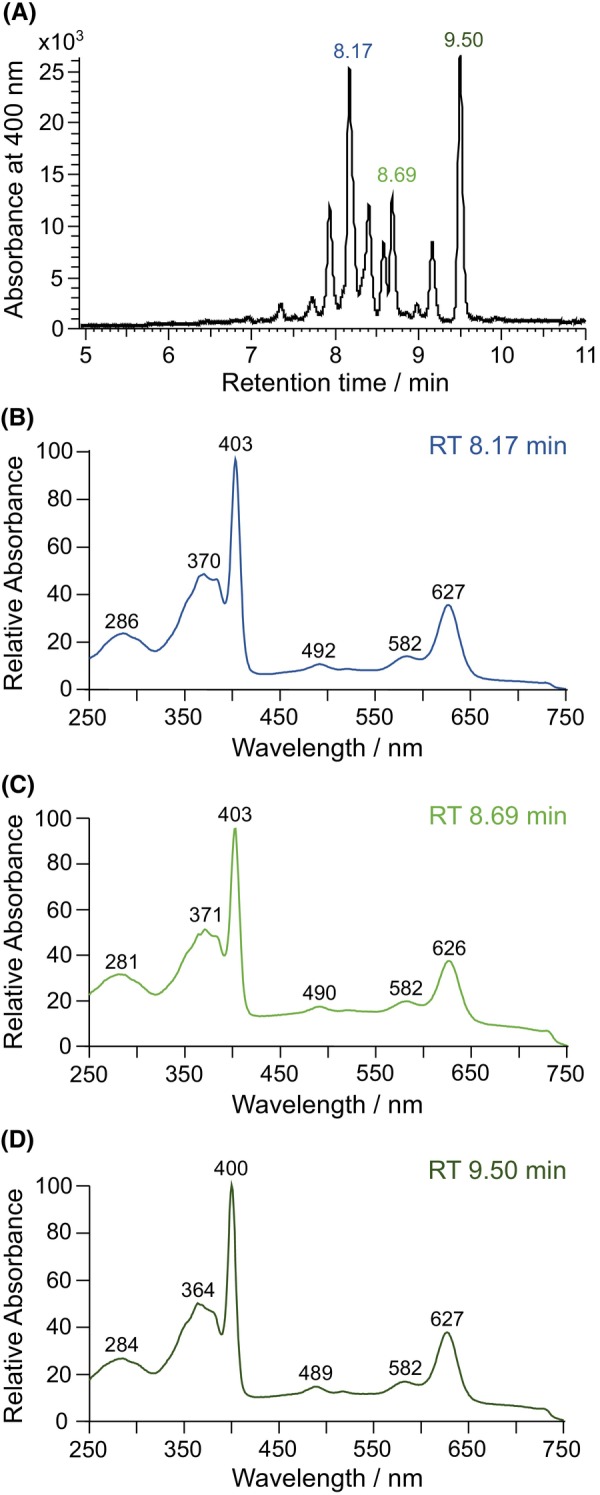
HPLC‐UV/Vis analysis of the NirJ activity assay after acid treatment. (A) HPLC‐UV/Vis analysis of a sample containing the products of a NirJ activity assay mixture (87 μm NirJ‐DDSH complex, 870 μm SAM, 870 μm DT) after 3 h of incubation and acid treatment under anaerobic conditions. (B–D) UV/Vis absorption spectra of the tetrapyrroles eluting at retention times of 8.17, 8.69, and 9.5 min, as indicated. The graphs were generated with originpro (version 2024; OriginLab Corporation) and freestyle (Thermo Fisher Scientific Inc., Waltham, MA, USA), and the figure was assembled using inkscape. RT, retention time.

**Fig. 7:**
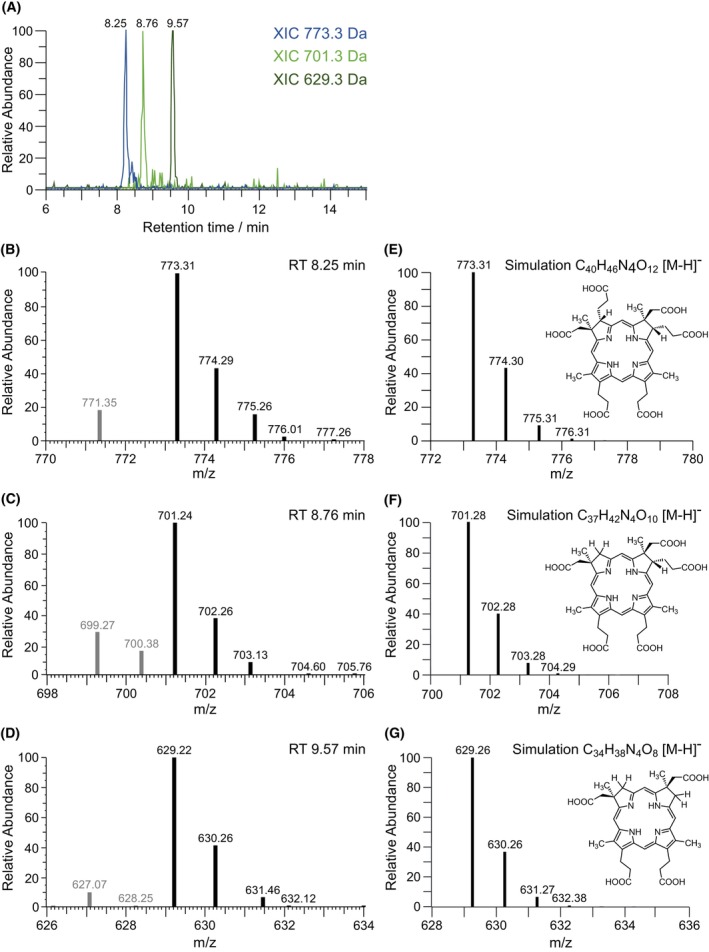
HPLC‐MS analysis of the NirJ activity assay after acid treatment. (A) Extracted ion chromatograms (XIC) at *m/z* of 773.3, 701.3, and 629.3, respectively, of the same sample as in Fig. 6. Retention times are indicated. (B–D) Mass spectra at retention times of 8.25 min (B), 8.76 min (C), and 9.57 min (D). (E–G) Simulated mass spectra for the [M‐H]^−^ ions of the tetrapyrroles shown on the right. Spectra were calculated with a free online tool at https://www.protpi.ch/Calculator/MassSpecSimulator. The graphs were generated with freestyle (Thermo Fisher Scientific Inc.), chemical structures were drawn with chemdraw (Revvity Signals Software Inc.) and the figure was assembled using inkscape. RT, retention time; XIC, extracted ion chromatogram.

In order to avoid the observed lactone formation and to identify the true reaction product of NirJ, efforts were made to keep the samples as anaerobic as possible after the NirJ activity assay and during the HPLC‐MS analysis. Therefore, HPLC solvents were made anaerobic and kept under nitrogen gas. Moreover, after the NirJ activity assay, tetrapyrroles were not extracted with ethyl acetate under aerobic conditions, but rather under anaerobic conditions using concentrated HCl for protein denaturation. The resulting tetrapyrrole solution showed a bluish fluorescence under UV light indicating that the central iron ion was removed, most likely due to the harsh treatment with strong acid. Nevertheless, this sample was analyzed by HPLC‐UV/Vis (Fig. 6) and HPLC‐MS (Fig. 7). In Fig. 6A, the HPLC‐UV/Vis chromatogram recorded at 400 nm is shown. Several peaks between 7.5 and 10 min are visible with major peaks at 8.17, 8.69, and 9.5 min. The UV/Vis absorption spectra (Fig. 6B–D) corresponding to the tetrapyrroles eluting at these retention times are similar to each other and resemble the spectra of sirohydrochlorin [19] and 12,18‐didecarboxy‐sirohydrochlorin [20], although there are differences in the absorption maxima compared to the spectra in the literature most likely due to the different solvents. Importantly, the observed UV/Vis spectra indeed indicate that the central iron ion of the tetrapyrroles was lost during acid extraction as stated above. In the HPLC‐MS chromatogram (electrospray ionization in negative ion mode), the retention times of all peaks are shifted by 0.07 min. The peaks at retention times of 8.25, 8.76, and 9.57 min correspond to substances with *m/z* of 773.3, 701.3, and 629.3, respectively, for the [M‐H]^−^, as indicated by the extracted ion chromatograms (XIC) at these masses (Fig. 7A). While the calculated mass of DDSH is 828.23 Da, the mass of 12,18‐didecarboxy‐sirohydrochlorin (DDSH lacking the central iron ion) is 774.31 Da. Therefore, the observed *m/z* of 773.3 for the peak at 8.25 min corresponds to the [M‐H]^−^ of 12,18‐didecarboxy‐sirohydrochlorin, the metal‐free form of the substrate of NirJ. Indeed, the observed mass spectrum at the retention time of 8.25 min corresponds to the simulated spectrum of 12,18‐didecarboxy‐sirohydrochlorin (Fig. 7B,E). The minor mass of 771.35 Da in the spectrum most likely represents small amounts of the monolactone form. The *m/z* of 701.3 and 629.3 at retention times of 8.76 and 9.57 min, respectively, are 72 and 144 Da less than the *m/z* of the [M‐H]^−^ of 12,18‐didecarboxy‐sirohydrochlorin. These differences are in line with the loss of one and two propionate side chains indicating that the corresponding substances represent the metal‐free intermediate and product of the NirJ reaction. Moreover, the observed *m/z* values for the [M‐H]^−^ ions are consistent with tetrapyrrole structures in which the propionate side chains were replaced by hydrogen resulting in methylene groups at positions C3 and C8 (Fig. 7C,D,F,G). Indeed, the observed mass spectra correspond to the respective simulated spectra. In the mass spectrum of the reaction intermediate, a small amount of the potential monolactone form with 699.27 Da is observed. Altogether, these results strongly suggest that the actual NirJ reaction product exhibits methylene groups at positions C3 and C8. This conclusion is substantiated by the fact that the UV/Vis absorption spectrum for the metal‐free NirJ reaction product observed in this study (Fig. 6D) is highly similar to the reported spectra of a synthetic methyl ester derivative [21] of the compound shown in Fig. 7G as well as of a synthetic octamethylisobacteriochlorin analogue [22]. Slight differences in the absorption maxima can be attributed to the different solvents used. A final proof for the proposed methylene groups at C3 and C8 could be provided by high‐resolution MS and NMR spectroscopy, both of which are technically challenging in the case of the NirJ reaction product due to the rapid formation of the lactone forms [6].

## Discussion

In this study, we showed that the aux‐cluster of NirJ is a [4Fe‐4S] cluster coordinated by four cysteine residues. Based on this finding, we exclude a role of the cluster in direct substrate binding, as there is no unoccupied coordination site. An alternative functional role for the aux‐cluster is electron transfer, as also proposed for the auxiliary clusters of other members of the radical SAM SPASM/twitch family [15, 23]. Indeed, the aux‐cluster of NirJ might function as an electron donor for the quenching of the intermediate radical, as elaborated in the following.

**Fig. 8:**
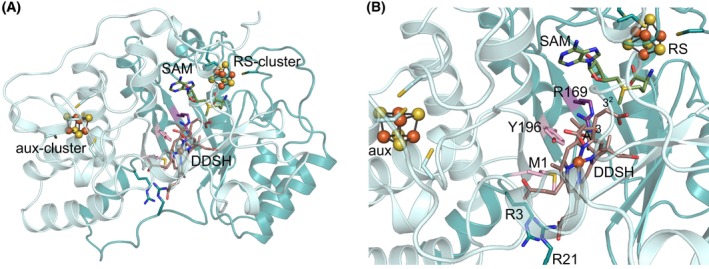
Computational model of NirJ. (A) Overall structure of NirJ containing both [4Fe‐4S] clusters (orange and yellow), cluster‐bound *S*‐adenosylmethionine (SAM, green) and the substrate 12,18‐didecarboxy‐siroheme (DDSH, dark salmon). The N‐terminal half of NirJ containing the partial TIM‐barrel domain characteristic for radical SAM enzymes is shown in dark teal, the C‐terminal half containing the SPASM/twitch domain is depicted in pale cyan. Cysteine residues coordinating the iron–sulfur clusters and potentially important amino acid residues within the active site are shown as sticks. (B) Closer view into the potential active site of NirJ. Amino acid residues discussed in the main text are labeled (numbering for NirJ from *Dinoroseobacter shibae*). Pyrrole ring A and positions C3 and C3^2^ of DDSH are indicated. The structural model was generated using chai‐1 [26], substrate docking was performed using webina [27] and the illustration was made using pymol (Version 3.0; Schrödinger, LLC, New York, NY, USA). The figure was assembled using inkscape. aux, auxiliary; DDSH, didecarboxy‐siroheme; SAM, *S*‐adenosyl‐l‐methionine; RS, radical SAM.

**Fig. 9:**
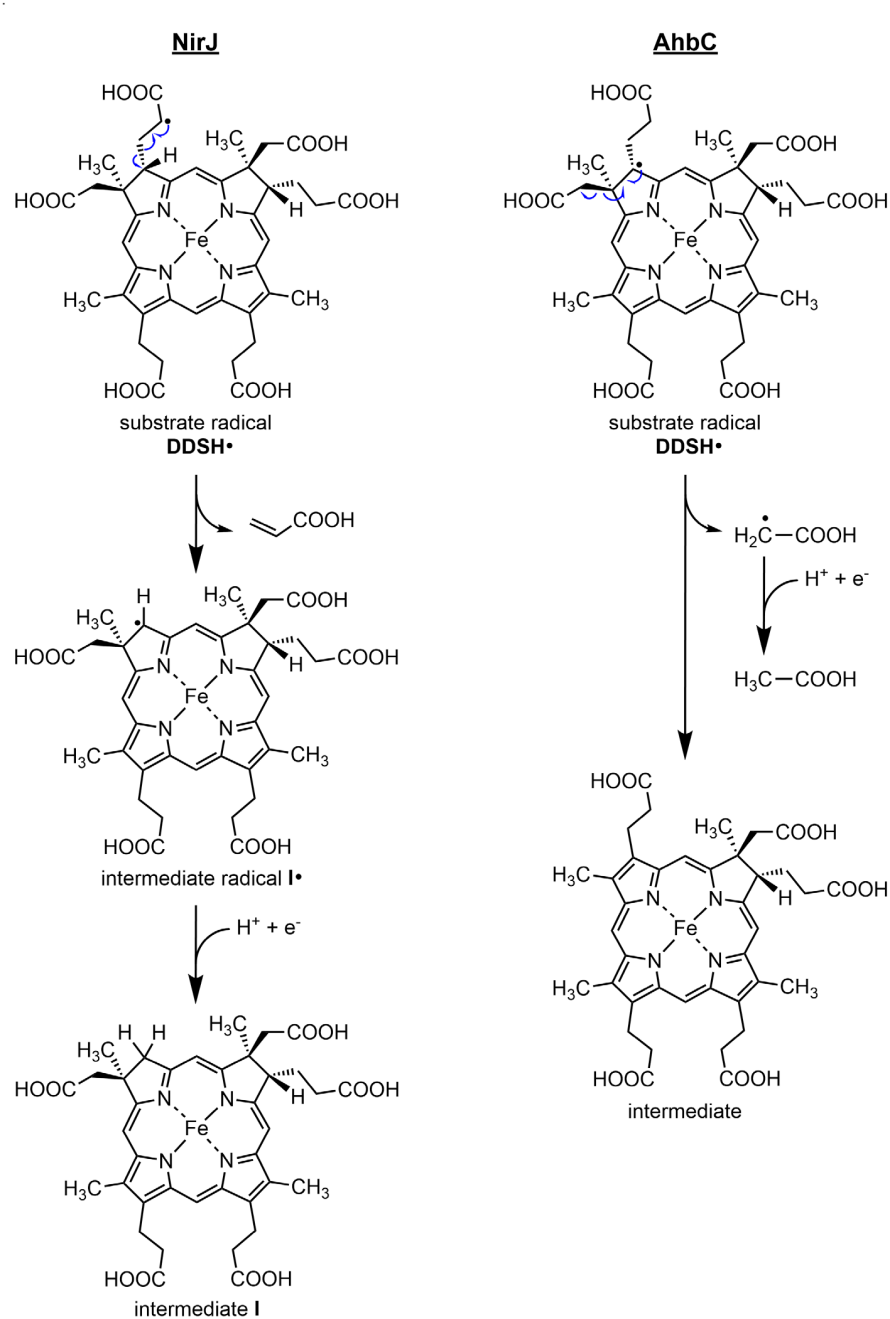
Radical reactions of the related enzymes NirJ and AhbC. On the left, the fragmentation of the substrate radical and the quenching reaction of the intermediate radical for the NirJ reaction are shown. As shown in this study, acrylic acid is released as a result of radical fragmentation and a methylene group at C3 is formed by radical quenching through addition of a proton and an electron. On the right, the proposed fragmentation of the substrate radical and the quenching reaction of the cleaved acetyl radical for the AhbC reaction are shown [4]. Note that for both enzymes the radical quenching step requires the addition of a proton and an electron. The electron donor might be the auxiliary iron–sulfur cluster. However, this hypothesis remains to be tested in the future. The figure was generated using chemdraw (Revvity Signals Software Inc.). DDSH, didecarboxy‐siroheme.

As described in the results section, we could show that acrylate is cleaved off as the leaving group leading to the formation of the intermediate radical shown in Fig. 1B. Based on the UV/Vis absorption and mass spectrum for the iron‐free derivative, we could also show that the final NirJ reaction product most likely carries methylene groups at positions C3 and C8. Therefore, the quenching of the intermediate radical could be achieved by the donation of an electron and a proton. Interestingly, radical quenching by transfer of an electron and a proton was also proposed for the radical SAM enzyme MftC with an auxiliary iron–sulfur cluster potentially acting as the electron donor [24]. Moreover, radical quenching through proton‐coupled electron transfer was also reported for MoaA, another member of the SPASM/twitch family, carrying an auxiliary [4Fe‐4S] cluster. In this case, an arginine residue was proposed to act as the proton donor and the aux‐cluster as the electron donor [25]. Based on these examples, we propose that in the case of NirJ the electron for radical quenching is provided by the reduced form of the aux‐cluster and the proton originates either from water or from a suitable amino acid residue within the active site of the enzyme. In order to further assess this hypothesis, a structural model of NirJ including both iron–sulfur clusters and SAM was generated using the Chai‐1 web interface [26]. Then, the substrate DDSH was roughly modeled into the potential active site using Webina 1.0.5 [27]. The resulting structural model reveals potential roles of several conserved amino acid residues (Fig. 8). For example, arginine residues Arg3 and Arg21 might be involved in substrate binding by coordinating the propionate carboxyl groups of pyrrole rings C and D. The side chains of methionine Met1 or tyrosine Tyr196 might coordinate the central iron ion of DDSH. Most importantly, the side chain of arginine residue Arg169 points towards C3 of pyrrole ring A, which is the site of the proposed intermediate radical. In the computational model, which was not further optimized, the distance between the guanidinium amino groups of Arg169 and C3 of DDSH is 4.0 Å. Thus, Arg169 might act as the proton donor required for radical quenching. Moreover, the distance of the aux‐cluster to the substrate DDSH (edge to edge) is 16.0 Å allowing for electron transfer. Altogether, external electron donors would be required for the reduction of the RS‐cluster as well as the aux‐cluster during NirJ catalysis. In the *in vitro* enzyme activity assay, dithionite serves as an artificial reductant for both clusters. With respect to the proposed radical quenching mechanism of NirJ, the close relationship between NirJ and AhbC should be mentioned (Fig. 9). AhbC is a radical SAM enzyme involved in the siroheme‐dependent heme biosynthesis pathway found in sulfate‐reducing bacteria and archaea, which catalyzes the removal of the two acetate side chains at positions C2 and C7 of DDSH to yield iron‐coproporphyrin III [4]. Thus, NirJ and AhbC use the same substrate. Strikingly, the amino acid sequences of NirJ and AhbC from different organisms share about 17% sequence identity and about 33% similarity, and AhbC also contains the C‐terminal cysteine motif CX_2_CX_5_CX_20–21_C. Therefore, it is reasonable to propose that AhbC might also contain an auxiliary [4Fe‐4S] cluster. Moreover, it was previously proposed that AhbC cleaves off the acetate side chains as acetyl radicals [4], the quenching of which could be achieved by the input of an electron and a proton. In this case, the proposed aux‐cluster could act as the electron donor in a similar fashion as we propose for the aux‐cluster in NirJ. However, the proposed role of the aux‐clusters of NirJ and AhbC as electron donating moieties has to be tested in the future including the determination of their redox potentials. Similarly, the identity of the proton donating amino acid residues remains to be determined for NirJ and AhbC.

Previous functional predictions concerning the role of NirJ in heme *d*_1_ biosynthesis included a scenario in which NirJ would also introduce the keto functions at positions C3 and C8 in addition to the removal of the propionate side chains [4]. Despite great efforts, we were never able to observe the formation of dihydro‐heme *d*_1_ in NirJ activity assays. Instead, in this study we provide evidence that NirJ forms a previously unknown pathway intermediate that lacks the oxo groups and most likely carries methylene groups at C3 and C8, which we name 3,8‐dideoxo‐dihydro‐heme *d*_1_ (IUPAC: [(2*R*,7*R*)‐2,3,7,8‐tetrahydro‐2,7‐bis(2‐hydroxy‐2‐oxo‐ethyl)‐3,7,12,18‐tetramethyl‐22*H*,24*H*‐porphin‐13,17‐dipropionato‐*N* ^21^,*N* ^22^,*N* ^23^,*N* ^24^]iron(II)). We postulate that 3,8‐dideoxo‐dihydro‐heme *d*_1_ is the actual NirJ product and that the keto functions are introduced by another enzyme, which remains to be identified. This conclusion is supported by the similarity between NirJ and AhbC, both catalyzing the removal of tetrapyrrole side chains with radical quenching steps requiring the input of electrons and protons, respectively, and not involving the introduction of oxo groups (Fig. 9).

## Materials and methods

### Chemicals

All chemicals and reagents were obtained from Carl Roth GmbH & Co. KG (Karlsruhe, Germany), Sigma‐Aldrich Chemie GmbH (Taufkirchen, Germany), or VWR International GmbH (Darmstadt, Germany). Nitrilotriacetic acid agarose was purchased from Macherey‐Nagel GmbH & Co. KG (Düren, Germany) [6]. ^57^Fe (96%) was obtained from Cyclotron Instruments (Mainz, Germany).

### Bacterial strains and plasmids

The *E. coli* strain DH10B was used for plasmid amplification, and *E. coli* BL21(DE3) was used as a host for recombinant protein production. Plasmids pAR8414 (here: pKK_*cysG*) [28] carrying the *cysG* gene from *E. coli* and pACYC_*nirDLGH*_*nirJ*‐His or pACYC_*nirDLGH*_*nirJ1CM*‐His encoding NirDLGH from *Pseudomonas aeruginosa* and NirJ or NirJ‐1CM from *Dinoroseobacter shibae* [6] were used for the simultaneous production of DDSH and NirJ or NirJ‐1CM. Plasmids pET22b_*nirJ* and pET22b_*nirJ1CM* [6] were employed for the production of NirJ and NirJ‐1CM without coproduction of DDSH.

### Production and purification of the NirJ‐DDSH complex

The production and purification of the NirJ‐DDSH complex was accomplished as described previously [6] with slight modifications. *E. coli* BL21(DE3) containing either plasmids pET22b_*nirJ*, pET22b_*nirJ1CM*, or plasmids pKK_*cysG* and pACYC_*nirDLGH*_*nirJ*‐His or pACYC_*nirDLGH*_*nirJ1CM*‐His together were cultivated at 37 °C in auto‐inducing medium ZYM5052 [29] supplemented with the appropriate antibiotics (100 μg·mL^−1^ ampicillin or 34 μg·mL^−1^ chloramphenicol) [6]. At an OD_600_ of 0.6–0.8, the cultures were shifted to 17 °C and further cultivated overnight (15–18 h) [6]. After cell harvest, the cell pellet was transferred into an anaerobic chamber containing 95% N_2_/5% H_2_ (Coy Laboratories, Grass Lake, MI, USA) [6] and suspended 1 : 1 (w/v) in anaerobic buffer containing 25 mm HEPES, pH 7.5, 200 mm NaCl, 30% glycerol (w/v), 25 mm imidazole.

Purification of the NirJ‐DDSH complex was performed under anaerobic conditions. The cells were disrupted by passing them through a pre‐cooled French® Pressure Cell at 1200 psi. The soluble protein fraction was obtained by centrifugation at 140 000 ***g*** for 1 h at 4 °C [6]. His_6_‐tagged NirJ was purified by immobilized metal ion affinity chromatography (IMAC) using a 1 mL gravity flow Ni‐nitrilotriacetic acid agarose column [6]. The soluble protein fraction was applied to the column equilibrated with 8 column volumes (CV) buffer A (25 mm HEPES, pH 7.5, 200 mm NaCl, 30% glycerol (w/v), 25 mm imidazole). Then, weakly and unspecifically bound proteins were removed by two washing steps, first with 4–8 CV of buffer W1 (25 mm HEPES, pH 7.5, 200 mm NaCl, 30% glycerol (w/v), 50 mm imidazole) and second with 2 CV of buffer W2 (25 mm HEPES, pH 7.5, 200 mm NaCl, 30% glycerol (w/v), 75 mm imidazole). The W2 fraction was collected, since a minor portion of the bound NirJ eluted during this washing step. The major portion of NirJ eluted with the elution buffer (25 mm HEPES, pH 7.5, 200 mm NaCl, 30% glycerol (w/v), 250 mm imidazole). After IMAC, the imidazole was removed from the NirJ elution fraction and the W2 fraction using a NAP™‐25 column (GE Healthcare, Freiburg, Germany) with buffer containing 25 mm HEPES, pH 7.5, 200 mm NaCl, and 30% glycerol (w/v). To increase the DDSH occupancy in NirJ of the main elution fraction, the NirJ of the W2 fraction was denatured via heat shock at 95 °C for 2 min. The denatured protein was then pelleted by centrifugation for 10 min at 14 000 ***g*** at room temperature. The supernatant containing the isolated DDSH was added to the main NirJ elution fraction and incubated for 5 min at room temperature in the dark. Unoccupied NirJ molecules immediately bind the free tetrapyrrole. If needed, the protein was concentrated with a 30 K Amicon® Ultracel centrifugal filter (Merck Millipore, Darmstadt, Germany) and directly used for further assays or stored at 4 °C [6].

### Preparation of NirJ samples for Mössbauer spectroscopy

The production and purification of NirJ and NirJ‐1CM for Mössbauer spectroscopy was accomplished in M9 minimal medium [30] with M9 minimal salts and trace elements supplemented with ^57^FeCl_3_ (50 μm), CaCl_2_ (0.1 mm), MgSO_4_ (2 mm) and 20% glucose (w/v). ^57^FeCl_3_ was prepared according to Ref. [31]. *E. coli* BL21(DE3) containing either plasmid pET22b_*nirJ* or pET22b_*nirJ1CM* was cultivated at 37 °C for 4–6 h, until an OD_600_ of 0.6–0.8 was reached. Protein production was induced with 0.4 mm IPTG, and cells were further incubated overnight (15–18 h) at 17 °C and 180 r.p.m. The cells were harvested by centrifugation, transferred into an anaerobic chamber (Coy Laboratories, Grass Lake, MI, USA) and suspended 1 : 1 (w/v) in anaerobic buffer containing 25 mm HEPES, pH 7.5, 200 mm NaCl, 30% glycerol (w/v), and 25 mm imidazole. Protein purification was performed under anaerobic conditions as described above.

For Mössbauer spectroscopy, the protein preparations were concentrated to a final volume of 300–450 μL. The final concentrations of the samples were 520 μm for ^57^Fe‐NirJ and 590 μm for ^57^Fe‐NirJ‐1CM. The samples were transferred to Mössbauer cups and frozen in liquid nitrogen.

### Mössbauer spectroscopy

Transmission Mössbauer spectra were recorded in constant acceleration mode using a conventional Mössbauer spectrometer (WissEl GmbH, Ortenberg, Germany). A 512‐channel analyzer was used in time‐scale mode. The 77 K spectra were measured using a flow cryostat (Oxford Instruments, Abingdon, UK). Field‐dependent Mössbauer spectroscopy was performed using a closed‐cycle helium cryostat (CRYO Industries of America Inc., Manchester, NH, USA) equipped with a self‐shielding superconducting magnet. The applied field of *B*_ext_ = 5 T was parallel to the γ‐rays [32]. For data analysis, the Microsoft Excel add‐on program Vinda was used [33]. The 77 K spectra were analyzed using Lorentzian line shapes. The Spin‐Hamilton formalism was used to simulate the field‐dependent spectra [34]. The isomer shifts were recorded relative to α‐iron at room temperature. Origin 2023 was used for graphical visualization of the spectra.

### Enzyme activity assay for HPLC–MS analysis of the NirJ product

Eighty‐seven micromolar NirJ‐DDSH (in buffer 25 mm Tris/HCl, pH 7.5, 200 mm NaCl, 10% glycerol (w/v)) was incubated anaerobically with 10 eq. SAM and DT for 3 h at room temperature in the dark. The reaction was stopped by the addition of 7 μL of 37% HCl to 100 μL assay mixture. The mixture was then centrifuged for 10 min at 14 000 ***g*** at room temperature. The supernatant was transferred to an HPLC vial with airtight crimp caps. The sample was stored anaerobically at 4 °C until anaerobic measurement using HPLC‐MS.

### Enzyme activity assay for HPLC‐UV/Vis analysis of NirJ product and derivatized cleavage byproduct

Two hundred micromolar NirJ‐DDSH were incubated anaerobically with 10 eq. SAM and DT, as well as 1 mm NaNO_2_ in a total volume of 1 mL at room temperature in the dark. One hundred microliter samples were withdrawn after 0 and 60 min, and the reaction was stopped by the addition of 2.5 μL of 12% HCl for the HPLC‐UV/Vis analysis of tetrapyrroles. The samples were stored at −20 °C until ethyl acetate extraction. For the derivatization of the cleavage byproduct, the remaining 800 μL of the reaction mixture was stopped after 60 min by the addition of acetonitrile at a ratio of 1 : 4 (v/v). The resulting mixture was frozen at −20 °C until derivatization.

### Enzyme activity assay for LC‐MS analysis of the derivatized cleavage byproduct

Three hundred and eighty micromolar NirJ‐DDSH was incubated anaerobically with 10 eq. SAM and DT, as well as 1 mm NaNO_2_ in a total volume of 1 mL at room temperature in the dark. One half of the reaction was stopped immediately (0 min) and the other half after 60 min of incubation by the addition of acetonitrile at a ratio of 1 : 4 (v/v). The same procedure was performed for a reaction mixture lacking the NirJ‐DDSH complex. The resulting mixtures were frozen at −20 °C until derivatization.

### Ethyl acetate extraction of tetrapyrroles

For tetrapyrrole extraction, 600 μL ethyl acetate was added to the stopped enzyme activity assay mixtures, and the samples were vortexed for 6 min. The precipitated protein and the aqueous phase were separated from the organic phase by centrifugation for 10 min at 21 000 ***g*** and 4 °C. The organic phase was carefully transferred to a new Eppendorf tube, and the solvent was evaporated to dryness in a Concentrator Plus vacuum centrifuge. The resulting pellet was reddish in color and stored at −20 °C until HPLC analysis.

### Derivatization of the NirJ byproduct

It was assumed that the decarboxylation byproduct of the NirJ reaction is a short‐chain carboxylic acid, possibly propionate or acrylate. HPLC analysis was facilitated by derivatization of the carboxylic acids, which was performed according to published procedures [35, 36] using propionate, acrylate, and methacrylate as standards. For the derivatization of the NirJ reaction byproduct, the stopped enzyme activity assay mixture was thawed, and the denatured protein was pelleted by centrifugation at 18 400 ***g*** and 4 °C for 10 min. The supernatant was transferred to a new Eppendorf tube, and excess acetonitrile was evaporated. The derivatization reagent (500 μL 1 m 1‐ethyl‐3‐(3‐dimethylaminopropyl)‐carbodiimid in acetonitrile, 2 μL *N*‐methylbenzylamine, 500 μL H_2_O) was then added at a ratio of 1 : 1 (v/v) and the reaction mixture was incubated for 1 h at 32 °C. The standard carboxylic acids were treated identically. The derivatized NirJ byproduct and standards were then extracted with ethyl acetate. For each 250 μL of the derivatization mixture, 800 μL ethyl acetate were added, and the samples were vortexed for 6 min. After centrifugation at 18 400 ***g*** and 4 °C for 10 min, the organic phase was transferred to a new Eppendorf tube, and the ethyl acetate was evaporated completely.

### HPLC‐UV/Vis analysis

HPLC‐UV/Vis analysis was performed at 25 °C using a JASCO HPLC 2000 series system (Jasco, Gross‐Umstadt, Germany) equipped with an MD‐2015 diode array detector [6].

For the analysis of extracted tetrapyrroles, the dried and frozen pellet was dissolved in 100 μL of the initial condition of the HPLC analysis (10 μL methanol, 10 μL acetonitrile, 80 μL 1 m ammonium acetate, pH 5.2). The separation of tetrapyrroles was accomplished as described before [6, 37, 38] with slight modifications. The column (Equisil BDS C18, 250 × 4.6 mm, 5 μm (Dr. Maisch HPLC GmbH, Ammerbuch‐Entringen, Germany)) was equilibrated with 80% 1 m ammonium acetate, pH 5.2 (solvent A), 10% methanol (solvent B) and 10% acetonitrile (solvent C) at a flow rate of 0.5 mL·min^−1^. After injection of 40 μL of sample, a linear gradient was run reaching 60% A, 30% B, 10% C after 25 min and 90% B, 10% C within the next 15 min. These conditions were held for 20 min before returning to the initial conditions [6]. The elution of tetrapyrroles was followed by recording the absorption at 385 and 400 nm.

The analysis of derivatized short‐chain carboxylic acids by HPLC‐UV/Vis was accomplished as described before [35, 36] with slight modifications. The column (Reprosil‐Pur C18‐AQ, 150 × 2 mm, 5 μm (Dr. Maisch HPLC GmbH)) was equilibrated with 70% of 0.1% aqueous formic acid (solvent A) and 30% acetonitrile (solvent B) at a flow rate of 0.2 mL·min^−1^. After the injection of 10 μL of sample (dried derivatized carboxylic acids dissolved in the initial conditions), the initial conditions were held for 25 min, after which the amount of solvent B was increased to 95% within 5 min. These conditions were held for another 10 min before returning to the initial conditions within 5 min. The elution of derivatized carboxylic acids was followed by recording the absorption at 272 nm.

### HPLC‐MS analysis

For the HPLC‐MS analysis of derivatized carboxylic acids, the dried samples (from activity assay and standards) were dissolved in a mixture of 20 μL acetonitrile and 80 μL of 0.1% aqueous formic acid. All samples were filtered through a 0.2 μm regenerated cellulose syringe filter (ISERA GmbH, Düren, Germany). The HPLC‐MS analysis was carried out on an Exion HPLC system (AB Sciex LLC, Framingham, MA, USA) connected to an ESI mass spectrometer (ABSciex Qtrap 5500) adjusted to the scan range of *m/z* 100–1500 in positive ion mode; target mass was set to *m/z* 150–200. The calculated masses of derivatized acrylate, propionate, and methacrylate are 175.1, 177.1, and 189.1 Da, respectively. Curtain gas pressure of 30 psi, ion spray voltage of 4000 V, and temperature of 400 °C were employed during analysis. Ion source gas 1 (GS1) was 40 psi, and ion source gas 2 (GS2) 50 psi. The declustering potential was 41 V. An Xbridge Premier BEH C18 column (150 × 2.1 mm, 2.5 μm (Waters, Milford, MA, USA)) was used at a flow rate of 0.5 mL·min^−1^. Solvent A was 0.1% aqueous formic acid, and solvent B was acetonitrile. The column was equilibrated with 80% A and 20% B. After 2 μL sample injection, these conditions were held for 35 min, after which the amount of solvent B was increased to 100% within 10 min. These conditions were held for another 10 min before returning to the initial conditions within 5 min. The elution of derivatized carboxylic acids was followed by recording the absorption at 272 nm and by MS.

The analysis of tetrapyrroles by HPLC‐MS and MS/MS was carried out on a Thermo Fisher Scientific HPLC UltiMate 3000 System TSQ Quantum Access Max mass spectrometer adjusted to the scan range of *m/z* 200–1500 in negative ion mode; the target mass was set to *m/z* 800. Curtain gas pressure of 30 psi, ion spray voltage of 2500 V, and a temperature of 450 °C were employed during the analysis. Sheath gas pressure was 30 psi, and aux gas pressure was 5 psi. The collision energy was 10 V. A Reprosil‐Pur C18‐AQ column (150 × 2 mm, 5 μm) was used at a flow rate of 0.5 mL·min^−1^. Solvent A was 0.1% aqueous formic acid (anaerobic) and solvent B was acetonitrile [6]. The column was equilibrated with 80% A and 20% B [6]. After 5 μL sample injection, these conditions were held for 3 min, after which the amount of B was increased to 100% within 17 min. These conditions were held for another 3 min before returning to the initial conditions. The elution of tetrapyrroles was followed by recording the absorption using a diode array detector and by MS.

## Author contributions

HM, MHH, and SJ planned and performed experiments. HM, MHH, KZ, SJ, VS, and GL analyzed data. HM and GL conceived the study. HM, MHH, VS, and GL wrote the manuscript.

## Conflict of interest

The authors declare no conflict of interest.

## Data Availability

The data that support the findings of this study are available from the corresponding author (gunhild.layer@pharmazie.uni-freiburg.de) upon reasonable request.
